# Supplementary material for: Dual HLA B*42 and B*81-reactive T cell receptors recognize more diverse HIV-1 Gag escape variants
Source: Nat Commun. 2018 Nov 27;9:5023. doi: 10.1038/s41467-018-07209-7 (PMC6258674; doi:10.1038/s41467-018-07209-7)
Supplement: Supplementary file 1 — Supplementary Information [file 41467_2018_7209_MOESM1_ESM.pdf]

Supplementary information

Dual HLA B*42 and B*81-reactive T cell receptors recognize more diverse HIV-1 Gag escape variants

Funsho Ogunshola[1,5,#], Gursev Anmole[2,#], Rachel L. Miller[3], Emily Goering[4], Thandeka Nkosi[1,5,], Daniel Muema[1], Jaclyn Mann[5], Nasreen Ismail[5], Denis Chopera[1], Thumbi Ndung'u[1,4,5,7], Mark A. Brockman[2,3,6,*], Zaza Ndhlovu[1,4,5,*]

[1]Africa Health Research Institute, University of KwaZulu-Natal, Durban, South Africa
[2]Department of Molecular Biology and Biochemistry, Simon Fraser University, Burnaby, BC V5A 1S6, Canada
[3]Faculty of Health Sciences, Simon Fraser University, Burnaby BC V5A 1S6, Canada
[4]Ragon Institute of MGH, MIT, and Harvard, Cambridge MA 02139, USA
[5]HIV Pathogenesis Programme, Doris Duke Medical Research Institute, University of KwaZulu-Natal, Durban, South Africa
[6]British Columbia Centre for Excellence in HIV/AIDS, Vancouver, BC V6Z 1Y6, Canada
[7]Max Planck Institute for Infection Biology, Berlin, Germany

[#]Contributed equally; *Corresponding authors and joint supervision

Supplementary Figure 1

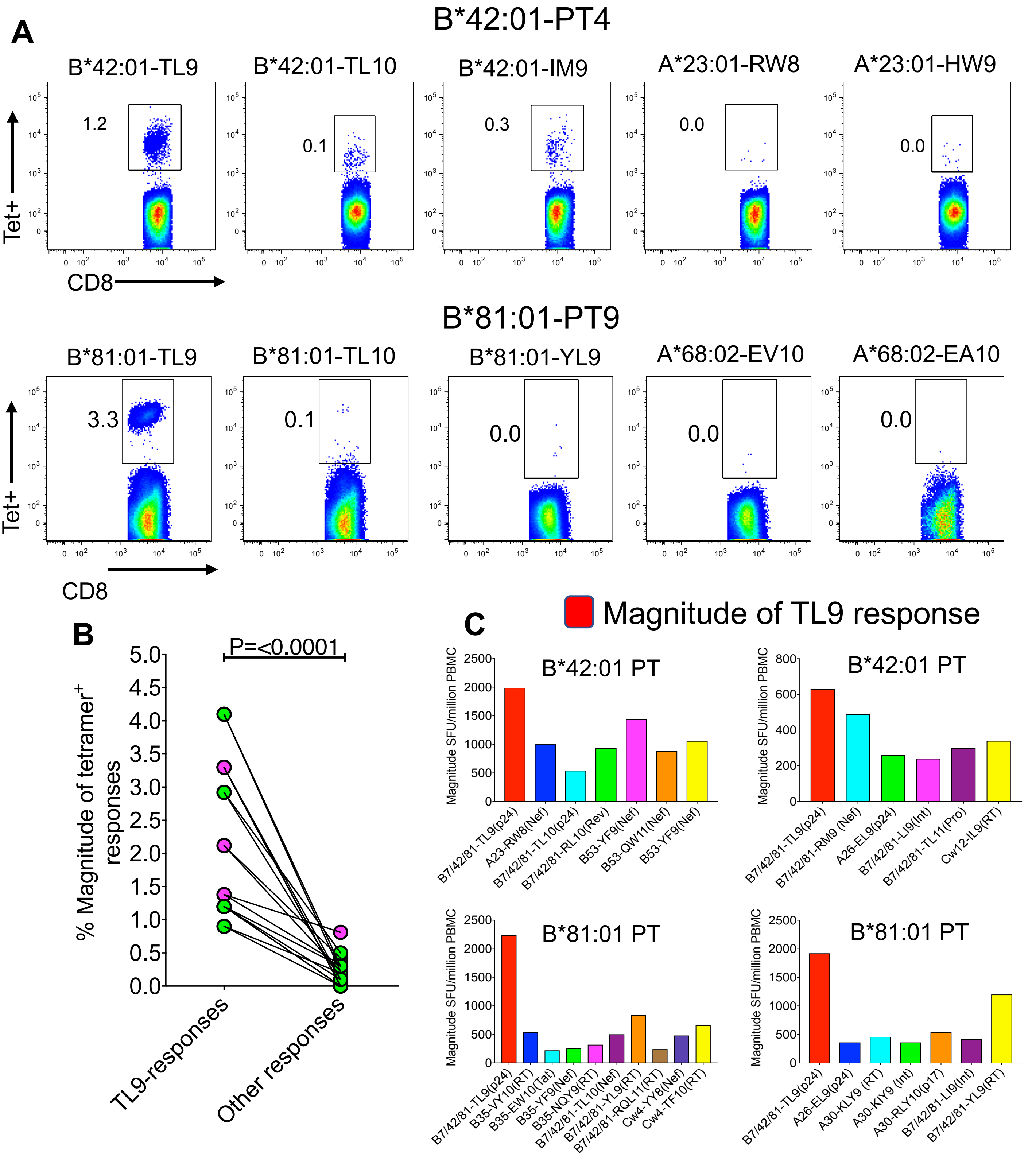

Intra-patient comparison of TL9 response with responses restricted by other alleles. Flow plot showing HIV-specific responses in a B*81:01 and B*42:01 representative donors (A), and aggregate data of TL9 responses compared to other responses (B) showing that TL9 responses are maintained at significantly higher frequencies than other responses, where green is B*42 and purple is B*81. ELISPOT data showing the magnitude of TL9 responses compared to other responses in B*81:01 and B*42:01 participants (C).

Supplementary Figure 2

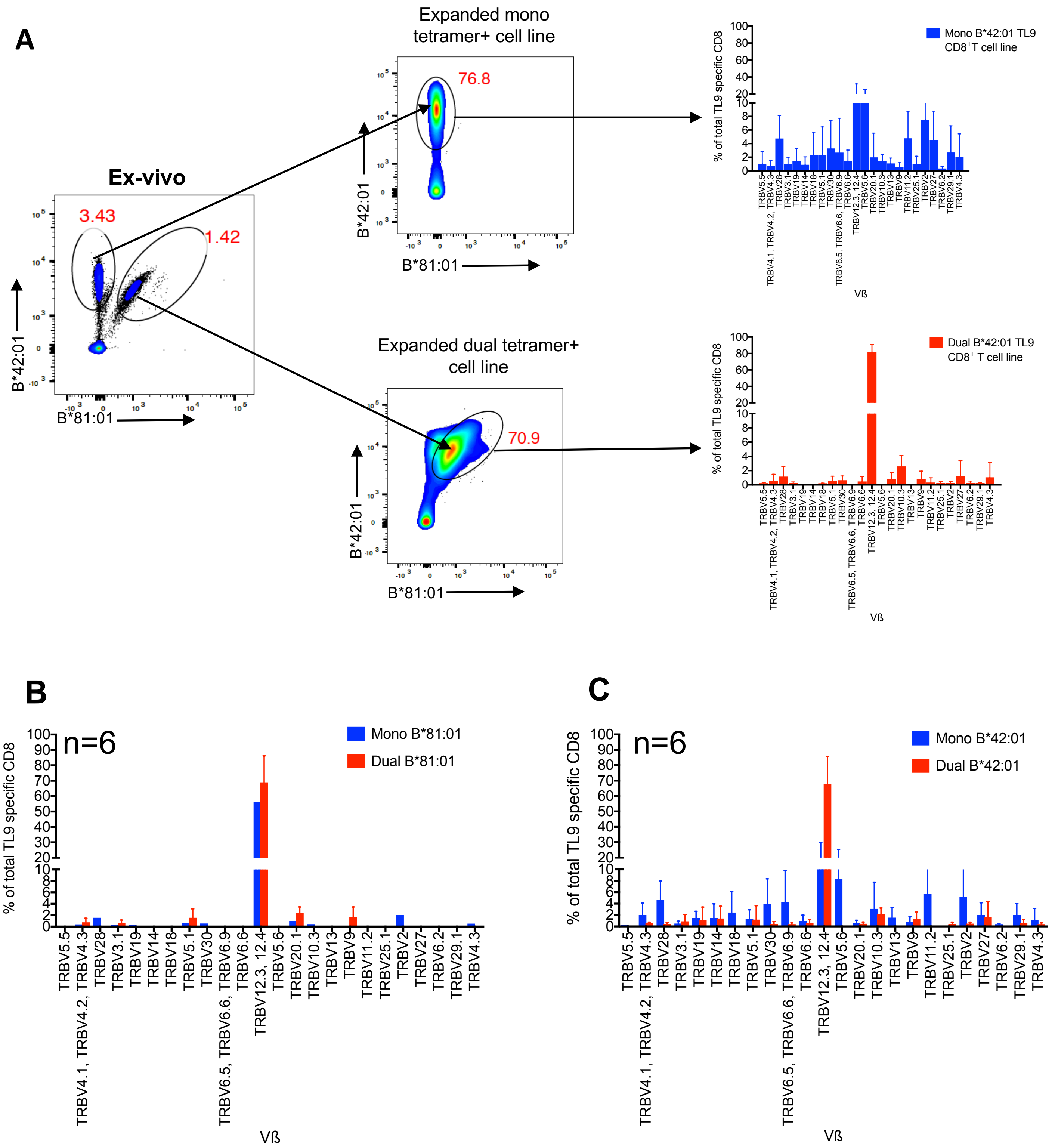

TCR-Vβ is conserved in dual TL9 tetramer⁺ CD8 T cell lines. Representative flow plot and TCR-Vβ family usage is shown for mono- and dual-reactive TL9 tetramer+ T cells isolated from a B*42:01 donor after expansion for 2 weeks (A). Aggregate data on TCR-Vβ family usage by mono-reactive compared to dual-reactive TL9 tetramer+ T cells in six B*81:01 donors (B) and six B*42:01 donors (C).

Supplementary Figure 3

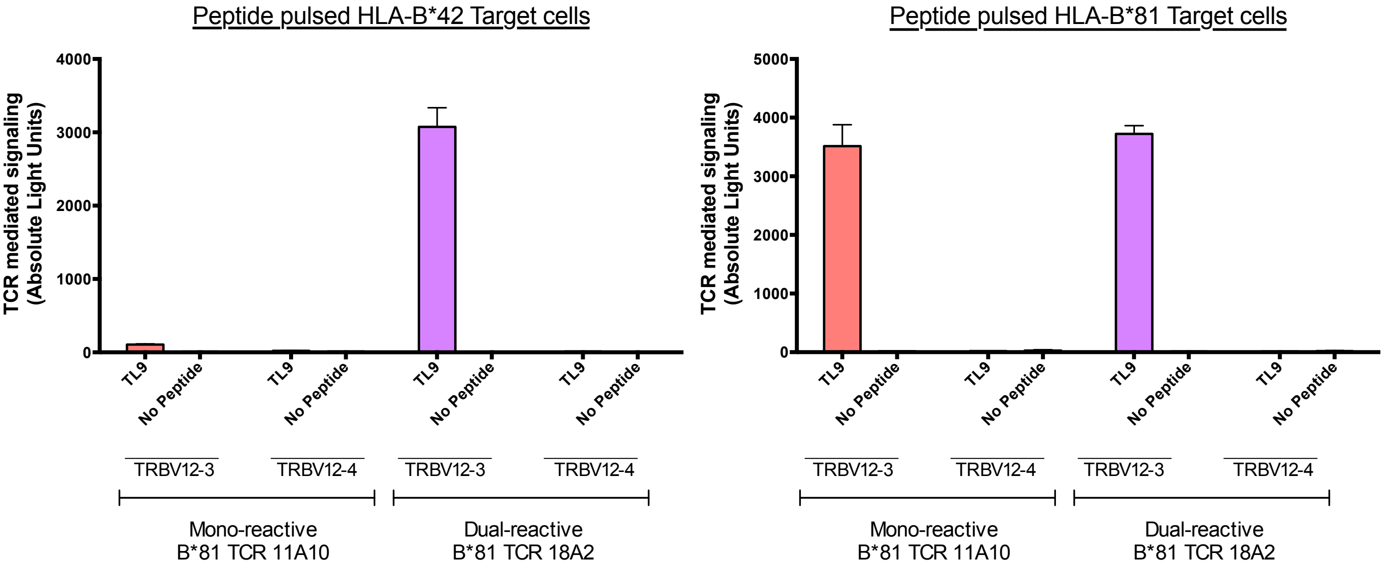

TRBV12-3 vs. TRBV12-4 signalling capacity. TCR were synthesized with TCR V beta genes 12-3 and 12-4 to assess functionality of the genes in the TCR reporter assay. Representative image indicated TRBV12-4 was non functional for the B*81 derived TCR 11A10 (red) and 18A2 (purple) at 20uM peptide concentration. Representative image where error bars indicate mean of 3 co-culture reactions, plus standard deviation. The experiment was conducted once to validate whether 12-3 or 12-4 are functional. As both TCR with 12-4 were non functional, we proceeded to order all TCR constructs with 12-3 for further experimentation.

Supplementary Figure 4

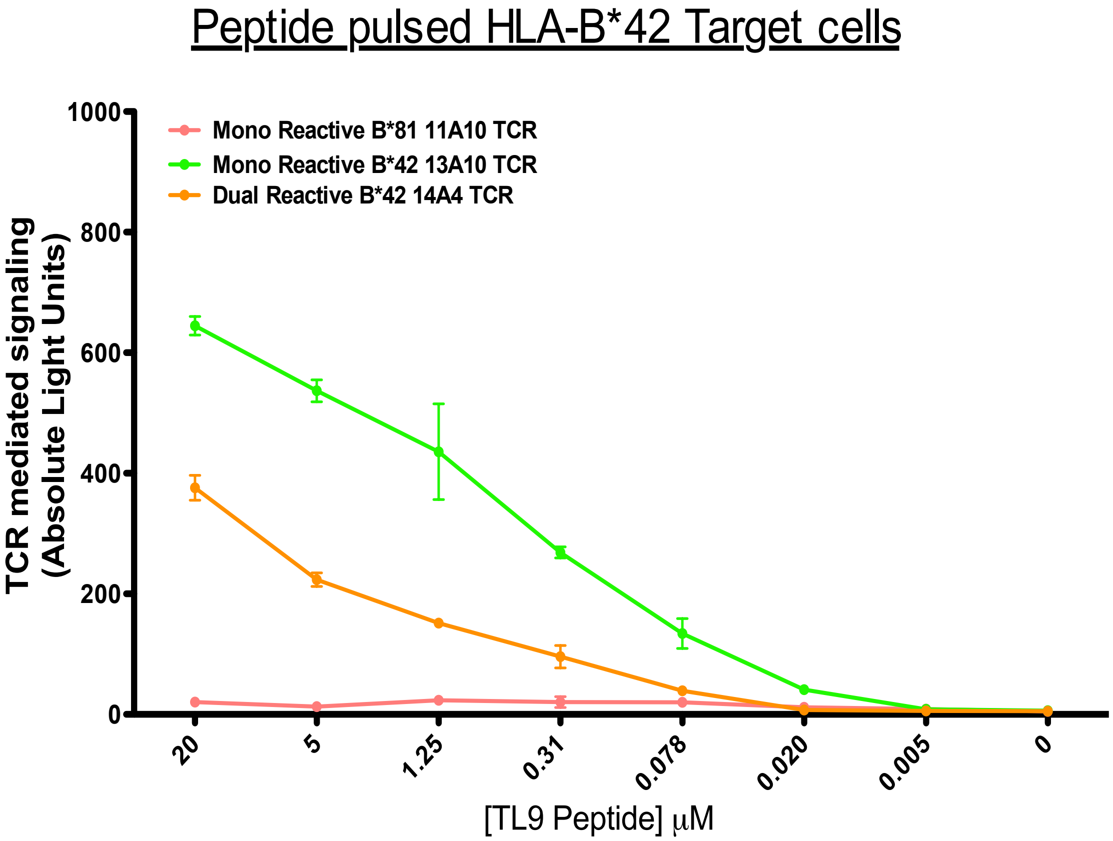

TCR signalling in response to TL9 peptide dilutions. Mono-reactive TCR clones 11A10 (B*81; red) and 13A10 (B*42; green) and dual-reactive TCR clone 14A4 (B*42; orange) were tested using target cells expressing HLA-B*42:01. Similar mono- or dual-reactive phenotypes were observed over a range of TL9 peptide doses (5 nM to 20 μM). The mono-reactive B*42:01-derived clone 13A10 displayed greater signalling activity compared to the dual-reactive clone 14A4 at all peptide doses tested. In addition, the mono reactive B*81:01-derived clone 11A10 was unable to recognize TL9 bound to HLA-B*42:01 at all peptide doses tested. Combined with data shown in Figure 4, these results confirm the mono- and dual-reactive phenotypes of these TCR clones and also suggest that antigen sensitivity is independent of dual-reactivity for the B*42:01-derived public TCR clones examined in this study. Representative image where error bars indicate mean of 3 co-culture reactions, plus standard error mean. The experiement was conducted 3 times.

Supplementary Table 1

Frequencies of HIV specific CD8^{+} T cell tetramer responses tested

PID	Class I HLA	Epitope tested	Tetramer response
**PT4**	A*23:01, A*29:02, B*53:01, B*42:01, C*03:04, C*17:00	**HLA-B*42:01 TL9**	**1.20**
		HLA-B*42:01 TL10	0.10
		HLA-B*42:01 IM9	0.30
		HLA-A*23:01 RW8	0.00
		HLA-A*23:01 HW9	0.00
**PT5**	A*30:01, A*34:02, B*35:01, B*42:01, C*02:10, C*17:01	**HLA-B*42:01 TL9**	**2.12**
		HLA-B*42:01 TL10	0.00
		HLA-B*42:01 IM9	0.00
		HLA-B*35:01 DL9	0.05
**PT6**	A*43:01, A*74:01, B*57:01, B*81:01, C*04:01, C*07:01	**HLA-B*81:01 TL9**	**2.08**
		HLA-B*81:01 TL10	0.00
		HLA-B*81:01 YL9	0.01
		HLA*C*07:01 KY11	0.53
**PT9**	A*23:01, A*68:02, B*14:02, B*81:01, C*08:02, C*18:00	**HLA-B*81:01 TL9**	**3.30**
		HLA-B*81:01 TL10	0.10
		HLA-B*81:01 YL9	0.00
		HLA-A*68:02 EV10	0.00
		HLA-A*68:02 EA10	0.00
**PT10**	A*02:05, A*33:01, B*42:01, B*15:03, C*07:01, B*17:01	**HLA-B*42:01 TL9**	**1.48**
		HLA-B*42:01 TL10	0.00
		HLA-B*15:03 FY10	0.81
		HLA-C*07:01 KY11	0.32
**PT13**	A*30:01, A*32:01, B*42:01, B*58:02, C*06:02, B*17:01	**HLA-B*42:01 TL9**	**4.70**
		HLA-B*42:01 TL10	0.00
		HLA-B*58:02 LF11	0.42
		HLA-B*58:02 QL11	0.30
**PT14**	A*02:01, A*30:01, B*42:01, B*45:07, C*16:01, B*17:01	**HLA-B*42:01 TL9**	**0.90**
		HLA-A*02:01 SL9	0.00
		HLA-B*42:01 TL10	0.20
		HLA-A*02:01 SV10	0.00
**PT15**	A*01:01, A*74:01, B*35:01, B*81:01, C*04:01, B*18:01	**HLA-B*81:01 TL9**	**1.38**
		HLA-B*42:01 TL10	0.10
		HLA-B*35:01 DL9	0.76
**PT18**	A*30:01, A*68:02, B*14:02, B*42:01, C*08:02, B*17:01	**HLA-B*42:01 TL9**	**4.60**
		HLA-B*42:01 TL10	0.10
		HLA-A*68:02 EA10	0.00
		HLA-A*68:02 EV10	0.20
**PT19**	A*23:01, A*30:01, B*42:01, B*57:02, C*07:01, B*17:00	**HLA-B*42:01 TL9**	**2.92**
		HLA-B*42:01 TL10	0.20
		HLA-B*57:02 TW10	0.10
		HLA-A*23:01 RW8	0.90
		HLA-C*07:01 KY11	0.50

All values are displayed as percent tetramer positive CD8^{+} T cells. Tetramer responses tested were based on published epitopes restricted by HLA alleles of the study participants and tetramer availability.

Supplementary Table 2

Forward

1st Round	Sequence
TRAV1-R1	CTGCACGTACCAGACATCTGGGTT
TRAV2,-R1	GGCTCAAAGCCTTCTCAGCAGG
TRAV3,-R1	GGATAACCTGGTTAAAGGCAGCTA
TRAV4,-R1	GGATACAAGACAAAAGTTACAAACGA
TRAV5,-R1	GCTGACGTATATTTTTTCAAATATGGA
TRAV6,-R1	GGAAGAGGCCCTGTTTTCTTGCT
TRAV7,-R1	GCTGGATATGAGAAGCAGAAAGGA
TRAV8,-R1	AGGACTCCAGCTTCTCCTGAAGTA
TRAV9,-R1	GTATGTCCAATATCCTGGAGAAGGT
TRAV10-R1	CAGTGAGAACACAAAGTCGAACGG
TRAV12.1,-R1	CCTAAGTTGCTGATGTCCGTATAC
TRAV12.2,-R1	GGGAAAAGCCCTGAGTTGATAATGT
TRAV12.3,-R1	GCTGATGTACACATACTCCAGTGG
TRAV13.1,-R1	CCCTTGGTATAAGCAAGAACTTGG
TRAV13.2,-R1	CCTCAATTCATTATAGACATTCGTTC
TRAV14,-R1	GCAAAATGCAACAGAAGGTCGCTA
TRAV16,-R1	TAGAGAGAGCATCAAAGGCTTCAC
TRAV17,-R1	CGTTCAAATGAAAGAGAGAAACACAG
TRAV18,-R1	CCTGAAAAGTTCAGAAAACCAGGAG
TRAV19,-R1	GGTCGGTATTCTTGGAACTTCCAG
TRAV20,-R1	GCTGGGGAAGAAAAGGAGAAAGAAA
TRAV21,-R1	GTCAGAGAGAGCAAACAAGTGGAA
TRAV22,-R1	GGACAAAACAGAATGGAAGATTAAGC
TRAV23,-R1	CCAGATGTGAGTGAAAAGAAAGAAG
TRAV24,-R1	GACTTTAAATGGGGATGAAAAGAAGA
TRAV25,-R1	GGAGAAGTGAAGAAGCAGAAAAGAC
TRAV26.1,-R1	CCAATGAAATGGCCTCTCTGATCA
TRAV26.2,-R1	GCAATGTGAACAACAGAATGGCCT
TRAV27,-R1	GGTGGAGAAGTGAAGAAGCTGAAG
TRAV29,-R1	GGATAAAAATGAAGATGGAAGATTCAC
TRAV30,-R1	CCTGATGATATTACTGAAGGGTGGA
TRAV34,-R1	GGTGGGGAAGAGAAAAGTCATGAA
TRAV35,-R1	GGTGAATTGACCTCAAATGGAAGAC
TRAV36,-R1	GCTAACTTCAAGTGGAATTGAAAAGA
TRAV38,-R1	GAAGCTTATAAGCAACAGAATGCAAC
TRAV39,-R1	GGAGCAGTGAAGCAGGAGGGAC
TRAV40,-R1	GAGAGACAATGGAAAACAGCAAAAAC
TRAV41,-R1	GCTGAGCTCAGGGAAGAAGAAGC

TRBV2,-R1	CTGAAATATTCGATGATCAATTCTCAG
TRBV3-1,-R1	TCATTATAAATGAAACAGTTCCAAATCG
TRBV4,-R1	AGTGTGCCAAGTCGCTTCTCAC
TRBV5-4,8,-R1	CAGAGGAAACTYCCCTCCTAGATT
TRBVS-1,-R1	GAGACACAGAGAAACAAAGGAAACTTC
TRBV6-1,-R1	GGTACCACTGACAAAGGAGAAGTCC
TRBV6-2,3,-R1	GAGGGTACAACTGCCAAAGGAGAGGT
TRBV6-4,-R1	GGCAAAGGAGAAGTCCCTGATGGTT
TRBV6-5,6,-R1	AAGGAGAAGTCCCSAATGGCTACAA
TRBV6-8,-R1	CTGACAAAGAAGTCCCCAATGGCTAC
TRBV6-9,-R1	CACTGACAAAGGAGAAGTCCCCGAT
TRBV7-2,-R1	AGACAAATCAGGGCTGCCCAGTGA
TRBV7-3,-R1	GACTCAGGGCTGCCCAACGAT
TRBV7-8,-R1	CCAGAATGAAGCTCAACTAGACAA
TRBV7-4,6,-R1	GGTTCTCTGCAGAGAGGCCTGAG
TRBV7-7,-R1	GGCTGCCCAGTGATCGGTTCTC
TRBV7-9,-R1	GACTTACTTCCAGAATGAAGCTCAACT
TRBV9,-R1	GAGCAAAAGGAAACATTCTTGAACGATT
TRBV10-1,3-R1	GGCTRATCCATTACTCATATGGTGTT
TRBV10-2,-R1	GATAAAGGAGAAGTCCCCGATGGCT
TRBV11,-R1	GATTCACAGTTGCCTAAGGATCGAT
TRBV12-3,4,-R1	GATTCAGGGATGCCCGAGGATCG
TRBV12-5,-R1	GATTCGGGGATGCCGAAGGATCG
TRBV13'-R1	GCAGAGCGATAAAGGAAGCATCCCT
TRBV14,-R1	TCCGGTATGCCCAACAATCGATTCT
TRBV15,-R1	GATTTTAACAATGAAGCAGACACCCCT
TRBV16,-R1	GATGAAACAGGTATGCCCAAGGAAAG
TRBV18,-R1	TATCATAGATGAGTCAGGAATGCCAAAG
TRBV19,-R1	GACTTTCAGAAAGGAGATATAGCTGAA
TRBV20-1,-R1	CAAGGCCACATACGAGCAAGGCGTC
TRBV24-1,-R1	CAAAGATATAAACAAAGGAGAGATCTCT
TRBV25-1,-R1	AGAGAAGGGAGATCTTTCCTCTGAGT
TRBV27-1,-R1	GACTGATAAGGGAGATGTTCCTGAAG
TRBV28,-R1	GGCTGATCTATTTCTCATATGATGTTAA
TRBV29,-R1	GCCACATATGAGAGTGGATTTGTCATT
TRBV30,-R1	GGTGCCCCAGAATCTCTCAGCCT

Reverse

1st Round	Sequence
TRAC,-R1	CGGTGAATAGGCAGACAGACTTGT
TRBC,-R1	ACCAGTGTGGCCTTTTGGGTGTG

1st round RT-PCR conducted with cocktail of forward and reverse primers. Primers have ability to encompass both TCR alpha and beta sequences. The concentration of each forward primer in the PCR reaction was 0.06 μM and the concentration of each reverse primer was 0.3 μM.

Supplementary Table 3

Forward

1st Round	Sequence
TRAV1,R2_TAG	CCAGGGTTTTCCCAGTCACGACAGGTCGTTTTTCTTCATTCCTTAGTC
TRAV2,R2_TAG	CCAGGGTTTTCCCAGTCACGACACGATACAACATGACCTATGAACGG
TRAV3.1,R2_TAG	CCAGGGTTTTCCCAGTCACGACCTTTGAAGCTGAATTTAACAAGAGCC
TRAV4.1,R2_TAG	CCAGGGTTTTCCCAGTCACGACCTCCCTGTTTATCCCTGCCGAC
TRAV5.1,R2_TAG	CCAGGGTTTTCCCAGTCACGACAAACAAGACCAAAGACTCACTGTTC
TRAV6,R2_TAG	CCAGGGTTTTCCCAGTCACGACAAGACTGAAGGTCACCTTTGATACC
TRAV7,R2_TAG	CCAGGGTTTTCCCAGTCACGACACTAAATGCTACATTACTGAAGAATGG
TRAV8,R2_TAG	CCAGGGTTTTCCCAGTCACGACGCATCAACGGTTTTGAGGCTGAATTTAA
TRAV9,R2_TAG	CCAGGGTTTTCCCAGTCACGACGAAACCACTTCTTTCCACTTGGAGAA
TRAV10,R2_TAG	CCAGGGTTTTCCCAGTCACGACTACAGCAACTCTGGATGCAGACAC
TRAV12,R2_TAG	CCAGGGTTTTCCCAGTCACGACGAAGATGGAAGGTTTACAGCACA
TRAV13.1,R2_TAG	CCAGGGTTTTCCCAGTCACGACGACATTCGTTCAAATGTGGGCGAA
TRAV13.2,R2_TAG	CCAGGGTTTTCCCAGTCACGACGGCAAGGCCAAAGAGTCACCGT
TRAV14,R2_TAG	CCAGGGTTTTCCCAGTCACGACTCCAGAAGGCAAGAAAATCCGCCA
TRAV16,R2_TAG	CCAGGGTTTTCCCAGTCACGACGCTGACCTTAACAAAGGCGAGACA
TRAV17,R2_TAG	CCAGGGTTTTCCCAGTCACGACTTAAGAGTCACGCTTGACACTTCCA
TRAV18,R2_TAG	CCAGGGTTTTCCCAGTCACGACGCAGAGGTTTTCAGGCCAGTCCT
TRAV19,R2_TAG	CCAGGGTTTTCCCAGTCACGACTCCACCAGTTCCTTCAACTTCACC
TRAV20,R2_TAG	CCAGGGTTTTCCCAGTCACGACGCCACATTAACAAAGAAGGAAAGCT
TRAV21,R2_TAG	CCAGGGTTTTCCCAGTCACGACGCCTCGCTGGATAAATCATCAGGA
TRAV22,R2_TAG	CCAGGGTTTTCCCAGTCACGACACGACTGTCGCTACGGAACGCTA
TRAV23,R2_TAG	CCAGGGTTTTCCCAGTCACGACCACAATCTCCTTCAATAAAAGTGCCA
TRAV24,R2_TAG	CCAGGGTTTTCCCAGTCACGACACGAATAAGTGCCACTCTTAATACCA
TRAV25,R2_TAG	CCAGGGTTTTCCCAGTCACGACGTTTGGAGAAGCAAAAAAGAACAGCT
TRAV26.1,R2_TAG	CCAGGGTTTTCCCAGTCACGACCAGAAGACAGAAAGTCCAGCACCT
TRAV26.2,R2_TAG	CCAGGGTTTTCCCAGTCACGACATCGCTGAAGACAGAAAGTCCAGT
TRAV27,R2_TAG	CCAGGGTTTTCCCAGTCACGACACTAACCTTTCAGTTTGGTGATGCAA
TRAV29,R2_TAG	CCAGGGTTTTCCCAGTCACGACCTTAAACAAAAGTGCCAAGCACCTC
TRAV30,R2_TAG	CCAGGGTTTTCCCAGTCACGACAATATCTGCTTCATTTAATGAAAAAAAGC
TRAV34,R2_TAG	CCAGGGTTTTCCCAGTCACGACCCAAGTTGGATGAGAAAAAGCAGCA
TRAV35,R2_TAG	CCAGGGTTTTCCCAGTCACGACCTCAGTTTGGTATAACCAGAAAGGA
TRAV36,R2_TAG	CCAGGGTTTTCCCAGTCACGACGGAAGACTAAGTAGCATATTAGATAAG
TRAV38,R2_TAG	CCAGGGTTTTCCCAGTCACGACCTGTGAACTTCCAGAAAGCAGCCA
TRAV39,R2_TAG	CCAGGGTTTTCCCAGTCACGACCCTCACTTGATACCAAAGCCCGT
TRAV40,R2_TAG	CCAGGGTTTTCCCAGTCACGACAGGCGGAAATATTAAAGACAAAAACTC
TRAV41,R2_TAG	CCAGGGTTTTCCCAGTCACGACGATTAATTGCCACAATAAACATACAGG

TRBV2,R2_TAG	CCAGGGTTTTCCCAGTCACGACGCCTGATGGATCAAATTTCACTCTG
TRBV3-1,R2_TAG	CCAGGGTTTTCCCAGTCACGACTCTCACCTAAATCTCCAGACAAAGCT
TRBV4,R2_TAG	CCAGGGTTTTCCCAGTCACGACCCTGAATGCCCCAACAGCTCTC
TRBV5-4,8,R2_TAG	CCAGGGTTTTCCCAGTCACGACCTCTGAGCTGAATGTGAACGCCT
TRBV5-1,R2_TAG	CCAGGGTTTTCCCAGTCACGACCGATTCTCAGGGCGCCAGTTCTCT
TRBV6-1,R2_TAG	CCAGGGTTTTCCCAGTCACGACTGGCTACAATGTCTCCAGATTAAACAA
TRBV6-2,3,R2_TAG	CCAGGGTTTTCCCAGTCACGACCCCTGATGGCTACAATGTCTCCAGA
TRBV6-4,R2_TAG	CCAGGGTTTTCCCAGTCACGACGTGTCTCCAGAGCAAACACAGATGATT
TRBV6-5,6,R2_TAG	CCAGGGTTTTCCCAGTCACGACGTCTCCAGATCAACCACAGAGGAT
TRBV6-8,R2_TAG	CCAGGGTTTTCCCAGTCACGACGTCTCTAGATTAAACACAGAGGATTTC
TRBV6-9,R2_TAG	CCAGGGTTTTCCCAGTCACGACGGCTACAATGTATCCAGATCAAACA
TRBV7-2,R2_TAG	CCAGGGTTTTCCCAGTCACGACTCGCTTCTCTGCAGAGAGGACTGG
TRBV7-3,R2_TAG	CCAGGGTTTTCCCAGTCACGACCGGTTCTTTGCAGTCAGGCCTGA
TRBV7-8,R2_TAG	CCAGGGTTTTCCCAGTCACGACCCAGTGATCGCTTCTTTGCAGAAA
TRBV7-4,6,R2_TAG	CCAGGGTTTTCCCAGTCACGACTCTCCACTCTGAMGATCCAGCGCA
TRBV7-7,R2_TAG	CCAGGGTTTTCCCAGTCACGACGCAGAGAGGCCTGAGGGATCCAT
TRBV7-9,R2_TAG	CCAGGGTTTTCCCAGTCACGACCTGCAGAGAGGCCTAAGGGATCT
TRBV9,R2_TAG	CCAGGGTTTTCCCAGTCACGACCTCCGCACAACAGTTCCCTGACTT
TRBV10-1,3,R2_TAG	CCAGGGTTTTCCCAGTCACGACCAGATGGCTAYAGTGTCTCTAGATCAAA
TRBV10-2,R2_TAG	CCAGGGTTTTCCCAGTCACGACGTTGTCTCCAGATCCAAGACAGAGAA
TRBV11,R2_TAG	CCAGGGTTTTCCCAGTCACGACGCAGAGAGGCTCAAAGGAGTAGACT
TRBV12-3,4,R2_TAG	CCAGGGTTTTCCCAGTCACGACGCTAAGATGCCTAATGCATCATTCTC
TRBV12-5,R2_TAG	CCAGGGTTTTCCCAGTCACGACCTCAGCAGAGATGCCTGATGCAACT
TRBV13,R2_TAG	CCAGGGTTTTCCCAGTCACGACTCTCAGCTCAACAGTTCAGTGACTA
TRBV14,R2_TAG	CCAGGGTTTTCCCAGTCACGACGCTGAAAGGACTGGAGGGACGTAT
TRBV15,R2_TAG	CCAGGGTTTTCCCAGTCACGACGATAACTTCCAATCCAGGAGGCCG
TRBV16,R2_TAG	CCAGGGTTTTCCCAGTCACGACGCTAAGTGCCTCCCAAATTCACCC
TRBV18,R2_TAG	CCAGGGTTTTCCCAGTCACGACGGAACGATTTTCTGCTGAATTTCCCA
TRBV19,R2_TAG	CCAGGGTTTTCCCAGTCACGACGGTACAGCGTCTCTCGGGAGAAGA
TRBV20-1,R2_TAG	CCAGGGTTTTCCCAGTCACGACGGACAAGTTTCTCATCAACCATGCAA
TRBV24-1,R2_TAG	CCAGGGTTTTCCCAGTCACGACTGGATACAGTGTCTCTCGACAGGC
TRBV25-1,R2_TAG	CCAGGGTTTTCCCAGTCACGACCAACAGTCTCCAGAATAAGGACGGA
TRBV27-1,R2_TAG	CCAGGGTTTTCCCAGTCACGACTACAAAGTCTCTCGAAAAGAGAAGAGGA
TRBV28,R2_TAG	CCAGGGTTTTCCCAGTCACGACGGGGTACAGTGTCTCTAGAGAGA
TRBV29,R2_TAG	CCAGGGTTTTCCCAGTCACGACGTTTCCCATCAGCCGCCCAAACCTA
TRBV30,R2_TAG	CCAGGGTTTTCCCAGTCACGACCAGACCCCAGGACCGGCAGTTCAT

Reverse

2nd Round	Sequence
TRAC,R2_TAG	AAGCAGTGGTATCAACGCAGAGTCAGACAGACTTGTCACTGGATTTAG
TRBC,R2_TAG	AAGCAGTGGTATCAACGCAGAGTCTTTTGGGTGTGGGAGATCTCTG

3rd Round Alpha reaction	Sequence
TCR_UNI_F	CCAGGGTTTTCCCAGTCACGAC
TRAC,-R2	CAGACAGACTTGTCACTGGATTTAG

3rd Round Alpha reaction	Sequence
TCR_UNI_F	CCAGGGTTTTCCCAGTCACGAC
TRBC,-R2	CTTTTGGGTGTGGGAGATCTCTG

2nd round PCR was conducted on 1 µL of RT-PCR product with cocktail of forward and reverse primers. Primers have ability to encompass both TCR alpha and beta sequences. The concentration of each forward primer in the second round PCR reaction was 0.06uM and the concentration of each reverse primer was 0.3uM. The second round PCR was diluted and 1uL was transferred into a 3rd round PCR, which was either TCR alpha or beta specific. With the respective 3rd round forward and reverse primers at a final concentration of 0.2 µM.
