# Supplementary material for: Dual HLA B*42 and B*81-reactive T cell receptors recognize more diverse HIV-1 Gag escape variants
Source: Nat Commun. 2018 Nov 27;9:5023. doi: 10.1038/s41467-018-07209-7 (PMC6258674; doi:10.1038/s41467-018-07209-7)
Supplement: Supplementary file 3 — Description of Additional Supplementary Files [file 41467_2018_7209_MOESM3_ESM.pdf]

Description of Additional Supplementary Files

Supplementary Data 1

Description: The supplementary data file includes all the normalized TCR signalling values from Figure 5 and relevant information to perform the analysis in Figure 6. This includes information on naturally occurring TL9 escapes variants, including frequency at the population level and translational mutations HIV-1 needs to pass through to reach escape mutation. Each TL9 variant and transitional mutant and its TCR recognition for all 8 TCR tested is also summarized in the data file for ease of reference.
